# Prevalence and Predictors of Vascular Cognitive Impairment in Patients With CADASIL

**DOI:** 10.1212/WNL.0000000000200607

**Published:** 2022-08-02

**Authors:** Amy A. Jolly, Stefania Nannoni, Hayley Edwards, Robin G. Morris, Hugh S. Markus

**Affiliations:** From the Stroke Research Group, Department of Clinical Neurosciences (A.A.J., S.N., H.E., H.S.M.), University of Cambridge, Cambridge Biomedical Campus, United Kingdom; and Department of Psychology (R.G.M.), King's College Institute of Psychiatry, Psychology and Neurosciences, Institute of Psychiatry, London, United Kingdom.

## Abstract

**Background and Objectives:**

Cerebral autosomal dominant arteriopathy with subcortical infarcts and leukoencephalopathy (CADASIL) is the most common monogenic form of stroke and early-onset dementia. We determined the prevalence of vascular cognitive impairment (VCI) in a group of patients with CADASIL and investigated which factors were associated with VCI risk, including clinical, genetic, and MRI parameters.

**Methods:**

Cognition was assessed in patients with genetically confirmed CADASIL (n = 176) and healthy controls (n = 265) (mean [SD] age 50.95 [11.35] vs 52.37 [7.93] years) using the Brief Memory and Executive Test (BMET) and the Montreal Cognitive Assessment (MoCA). VCI was defined according to previously validated cutoffs. We determined the prevalence of VCI and its associations with clinical risk factors, mutation location (epidermal growth factor–like repeats [EGFr] 1–6 vs EGFr 7–34), and MRI markers of small vessel disease.

**Results:**

VCI was more common in patients with CADASIL than in controls; 39.8 vs 10.2% on the BMET and 47.7% vs 19.6% on the MOCA. Patients with CADASIL had worse performance across all cognitive domains. A history of stroke was associated with VCI on the BMET (OR 2.12, 95% CI [1.05, 4.27] *p* = 0.04) and MoCA (OR 2.55 [1.21, 5.41] *p* = 0.01), after controlling for age and sex. There was no association of VCI with mutation site. Lacune count was the only MRI parameter independently associated with VCI on the BMET (OR: 1.63, 95% CI [1.10, 2.41], *p* = 0.014), after controlling for other MRI parameters. These associations persisted after controlling for education in the sensitivity analyses.

**Discussion:**

VCI is present in almost half of the patients with CADASIL with a mean age of 50 years. Stroke and lacune count on MRI were both independent predictors of VCI on the BMET.


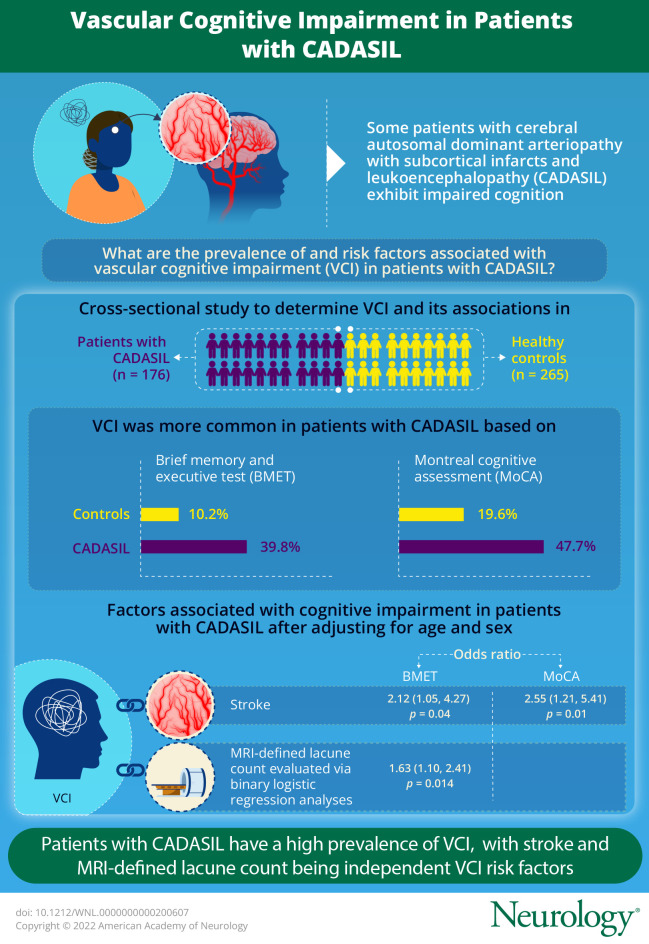


Cerebral autosomal dominant arteriopathy with subcortical infarcts and leukoencephalopathy (CADASIL) is the most common inherited form of stroke.^1^ Mutations in the *NOTCH3* gene result in small vessel arteriopathy, characterized clinically by early-onset stroke, vascular cognitive impairment (VCI), and dementia.^1-4^ Although most symptomatic patients will exhibit some cognitive impairment in later years,^3-6^ the disease course and severity are very variable between individuals and even within families.^1,3^ The reason for this is uncertain, although vascular risk factors have been associated with more rapid progression,^1,2,7^ and recently, the mutation site has been associated with severity, with more proximal mutations resulting in earlier stroke onset.^8^ Previous studies have shown that early features of cognitive impairment in CADASIL include executive dysfunction and slowing of information processing speed.^1,9-14^ Early effects on working memory and episodic long-term memory have been less consistently associated.^5,9,12,13^ Most previous studies of cognition in CADASIL have been small or moderate in size.

In a large group of patients with CADASIL, we determined the pattern and prevalence of cognitive impairment. We then determined which factors, including mutation location, were associated with the cognitive impairment and how cognitive impairment related to MRI markers of the disease including lacunar infarcts, white matter hyperintensities (WMH), and brain atrophy.

## Methods

### Study Population

#### Patients With CADASIL

In this analysis, patients with CADASIL were included from a national CADASIL clinic in Cambridge, UK, and as part of the UK Familial Cerebral Small Vessel Disease (SVD) study, which recruits patients with monogenic SVD from 6 neurologic centers in the UK (see acknowledgements). Information was collected prospectively on clinical presentation, vascular risk factors, and family history, and original clinical brain MRI images were obtained. We included the first 265 patients with CADASIL recruited to the study. Of them, 176 had cognitive assessments available and were included in this analysis. All patients had typical cysteine changing mutations.

### Control Participants

Healthy controls were previously included as part of the Brief Memory and Executive Test (BMET) validation study by Brookes et al.^14^ This consisted of 502 healthy volunteers with no history of stroke, for whom vascular risk factors and demographic information were collected and neuropsychological testing was administered. A total of 265 individuals were selected to roughly match the age and sex distribution of the patients, as seen on histogram.

### Neuroimaging Features

Original brain MRIs of patients with CADASIL, acquired as part of routine clinical assessment, were reviewed by a neurologist (S.N.). Fluid-attenuated inversion recovery and T1 sequences were reviewed to determine the presence and number of old cavitated lacunar infarcts and diffusion-weighted imaging sequences to identify recent lacunar infarcts. A lacunar infarction was defined as a subcortical infarct between 3 and 15 mm in diameter.^15^ Cerebral microbleeds (CMBs) were graded on gradient echo (GE) or susceptibility-weighted imaging (SWI) sequences using the Brain Observer Microbleed Rating Scale.^16^ Brain volume, while adjusting for skull size, was estimated using SIENAX (from FSL software, fsl. fmrib.ox.ac.uk,^17^) with T1 sequences. SIENAX first extracts brain tissue, then estimates brain volume.^18^ White matter hyperintensities (WMH) were defined as areas of increased signal on fluid-attenuated inversion recovery images and quantified by trained raters, using a semiautomated contouring tool in the Jim image analysis software, version 8 (Xinapse Systems, xinapse.com/j-im-8-software/). Interrater agreement for WMH lesion volume was calculated in a subset of data (n = 10) and showed good agreement (intraclass correlation coefficient = 0.96).

### Neuropsychological Testing

#### Brief Memory and Executive Test

The Brief Memory and Executive Test (BMET) is a cognitive screening tool designed and previously validated to be sensitive to the cognitive deficit seen in sporadic SVD and shown to be sensitive to the cognitive deficit in CADASIL.^14,19,20^ The test comprises 8 tasks (domains) that provide measures that form 2 subscales and an overall score. The first subscale, Executive Functioning and Processing Speed, is calculated using the tasks Letter-Number Matching, Motor Sequencing, Letter Sequencing, and Number-Letter Sequencing.^20^ The second subscale, Orientation and Memory, is made up of the Orientation, 5-item Repetition, 5-item Recall, and 5-item Recognition tasks.^20^ The measures from each task are transformed into scales that have a maximum of 2, giving a maximum total of 8 on each subscale and 16 overall.^20^ These scorings are age-adjusted, with each measure adjusted for the age of the participant.^20^ The BMET is freely available to download (bmet.info).

### Montreal Cognitive Assessment

The Montreal Cognitive Assessment (MoCA) is a brief cognitive screening tool for detecting mild cognitive impairments.^21^ The MoCA has 8 domains: visuospatial, naming, memory, attention, language, abstraction, delayed recall, and orientation, aimed overall at measuring global cognition.^21^

### Standard Protocol Approvals, Registrations, and Patient Consents

All participants gave written informed consent. For those without capacity, a consultee gave written informed consent. The UK Familial SVD study was approved by the East of England Cambridge Central Research Ethics Committee (16/EE/0118). Control data collection study was approved by the London Bridge Research Ethics Committee (11/LO/0636).

### Statistical Analyses

Comparison of demographics and vascular risk factors between CADASIL patients with and without stroke was assessed using the independent *t-*tests, χ^2^ tests, and logistic regression, where appropriate.

The mean values and SDs on the BMET and the MoCA and it's measures and scales were calculated. To examine the cognitive profile of the groups, the mean values and standard deviations of the control group were used to calculate the *z* scores for the overall CADASIL group and for those with and without stroke in the CADASIL group.^11^ Z scores for the control group were by definition set at zero.^11^ VCI on the BMET was defined using a previously validated cutoff of ≤13 on the overall score.^20^ On the MoCA, patients were classified as having VCI when scoring ≤25 of 30 for the total score, as previously defined.^21^

Performance on the BMET was compared between patients with CADASIL and controls and between patients with CADASIL subdivided into those with and without a history of stroke, using the Mann-Whitney *U* test. Comparison of performance on the MoCA was determined using analysis of covariance to assess performance on the MoCA and its subscales, with age as a covariate because MoCA is not age adjusted. Binary logistic regression was used to determine whether clinical features and risk factors predicted VCI on the BMET and MoCA.

Owing to their non-normal distribution, MRI parameters were normalized. Normalized WMH lesion volume (accounting for skull size) and lacune count were normalized using square root transformation. Normalized total brain volume (NBV) (again, accounting for skull size) was normalized using a square transformation. Microbleed count (CMB) was normalized with logarithm transformation. Binary logistic regression was run within the CADASIL group to determine whether MRI parameters predicted VCI on the BMET and MoCA. Because only a subgroup had sequences to allow quantification of CMB, microbleeds were excluded from this analysis and assessed separately. Mutation site was categorized as a binary variable by dividing the NOTCH3 protein's 34 epidermal growth factor–like repeats (EGFrs) into 2 groupings: EGFr 1–6 and EGFr 7–34, as previously described.^8^ Binary logistic regression was run within the CADASIL group to determine whether mutation site predicted VCI on the BMET and MoCA.

Education was assessed as a binary variable, with ≤12 years of education as a cutoff, in line with the MoCA.^21^ Owing to some of the CADASIL group having missing years of education data (n = 61), analyses were initially run without education as a covariate. An additional sensitivity analysis including education as a covariate was then conducted on all analyses, separately. Missing values were coded as such using −99 to retain sample size.

### Data Availability

On reasonable request, data from this study are available from the corresponding author.

## Results

Demographics for patients with CADASIL and controls are summarized in Table 1. Hypertension and hypercholesterolemia were more common in patients with CADASIL than in controls. More than 12 years education was more common in controls.

**Table 1:**
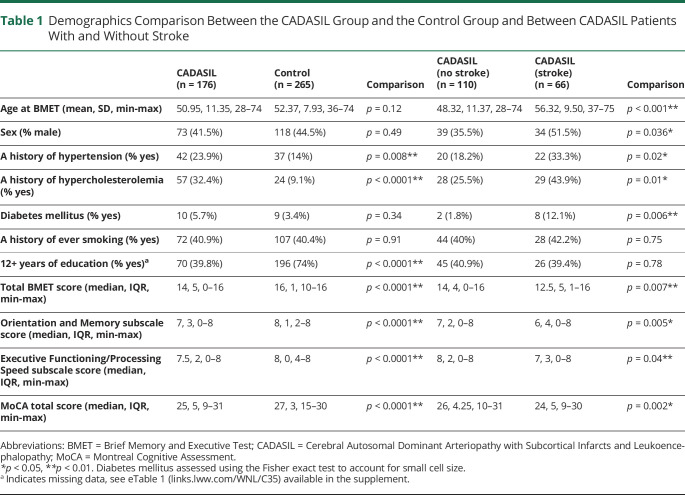
Demographics Comparison Between the CADASIL Group and the Control Group and Between CADASIL Patients With and Without Stroke

### Cognition Performance Between Groups

**Figure 1:**
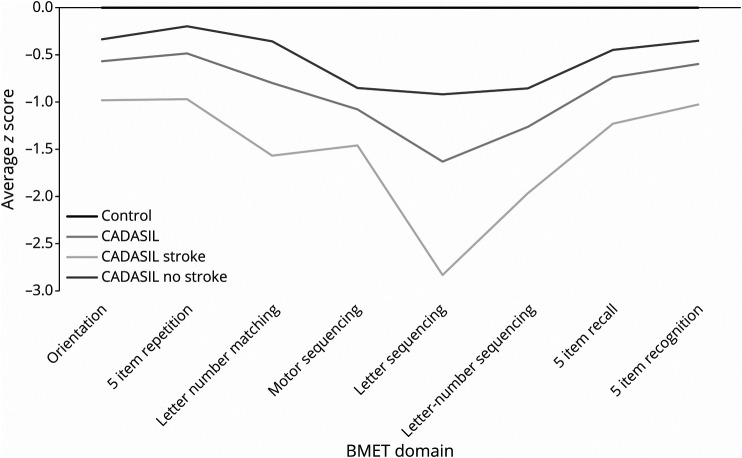
Z Scores of Cerebral Autosomal Dominant Arteriopathy with Subcortical Infarcts and Leukoencephalopathy Patients Overall, With and Without Stroke and Controls on the Individual Brief Memory and Executive Test Tasks

**Figure 2:**
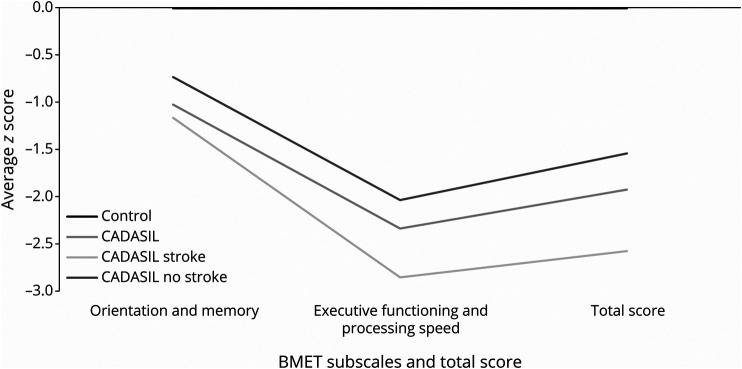
Z Scores of Cerebral Autosomal Dominant Arteriopathy with Subcortical Infarcts and Leukoencephalopathy Patients Overall, With and Without Stroke and Controls on the BMET Subscales and Total Brief Memory and Executive Test Score

On the BMET, patients with CADASIL had significantly worse performance than controls on total BMET score (Mann-Whitney, *p <* 0.0001), Orientation and Memory subscale score (Mann-Whitney, *p <* 0.0001), and Executive Functioning and Processing Speed subscale score (Mann-Whitney, *p <* 0.0001) (Table 1). They also performed worse on total MoCA score in age-adjusted analysis (*p* < 0.0001) (Table 1). Z score plots show the pattern of impairment across all BMET individual measures (Figure 1) and on the BMET subscales and total score (Figure 2); Patients with CADASIL had lower performance across all domains, with the greatest impairment on the Letter Sequencing task (Figure 1)

VCI, defined using the BMET, was present in 39.8% of the CADASIL group and 10.2% of controls. VCI, defined using the MoCA, was present in 47.7% of the CADASIL group and 19.6% of controls. On multivariate analysis, controlling for age and sex, VCI was more common in patients with CADASIL when VCI was defined on both on the BMET (OR = 6.39, 95% CI [3.85, 10.62], *p* < 0.001) and MoCA (OR = 4.23, 95% CI [2.69, 6.65], *p* < 0.001). Sensitivity analysis controlling for education showed the relationship remained significant on the BMET (*p* < 0.001) and on the MoCA (*p* < 0.001).

CADASIL patients with stroke were more likely to be male, older, and experience hypertension, hypercholesterolemia, and diabetes compared with CADASIL patients without stroke (Table 1). CADASIL patients with stroke had worse performance on total BMET score (*p* = 0.007), Executive Functioning and Processing subscale score (*p* = 0.04), and Orientation and Memory subscale score (*p* = 0.005) than stroke-free CADASIL patients (Table 1). This impairment was seen across all cognitive domains (Figures 1 and 2). There was also a significant difference in total MoCA between the 2 CADASIL groups *(p* = 0.002) (Table 1).

### Risk Factors for the Presence of VCI

Factors associated with VCI as determined on either the BMET or MoCA are summarized in Table 2. Age was associated with VCI on the MoCA but not on the BMET, which uses age-adjusted norms. On age-adjusted and sex-adjusted analysis, smoking was associated with decreased VCI on the MoCA, and stroke was associated with increased VCI on both the BMET and MoCA (Table 2).

**Table 2:**
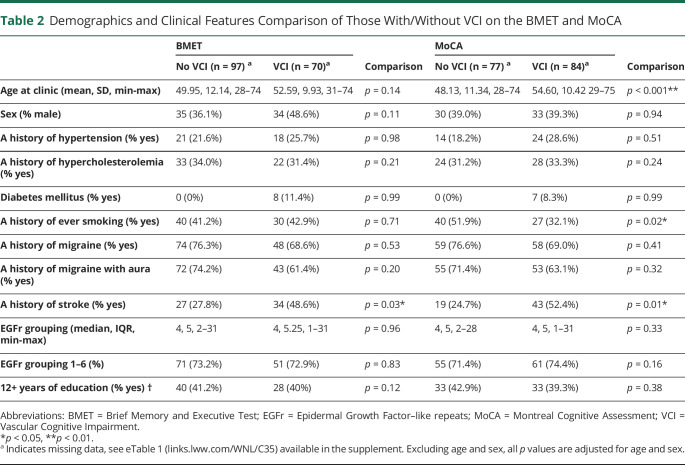
Demographics and Clinical Features Comparison of Those With/Without VCI on the BMET and MoCA

While controlling for age and sex, only stroke (OR 2.12, 95% CI [1.05, 4.27] *p* = 0.04) was associated with increased VCI on the BMET. On multivariate analyses, looking at a history of stroke and a history of migraine with aura, age (OR 1.04, 95% CI [1.01, 1.08] *p* = 0.01) and a history of stroke (OR 2.55, 95% CI [1.21, 5.41] *p* = 0.01) were associated with increased VCI on MoCA, while controlling for age and sex. These relationships remained significant when controlling for education in the sensitivity analyses: MoCA (stroke, *p* = 0.02; Age, *p* = 0.008), BMET (stroke, *p* = 0.03). While controlling for age and sex, there was no relationship between mutation site (EGFr 1–6 vs EGFr 7–34) and VCI on the BMET or MoCA (*p* = 0.83 and *p* = 0.58, respectively).

### Relationship Between MRI Parameters, Cognition, and the Presence of VCI

Within the CADASIL group, while controlling for age and sex, lacune count was significantly and negatively correlated to total BMET score (r_s_ = −0.266, *p* = 0.001) and both Executive Functioning and Processing Speed (r_s_ = −0.224, *p* = 0.007) and Orientation and Memory subscales (r_s_ = −0.218, *p* = 0.009). In the sensitivity analysis, these remained significant when controlling for education: *p* < 0.001, *p* = 0.005, and *p* = 0.007, respectively.

By contrast, there was no difference in WMH lesion volume, CMB count, and normalized brain volume between those with and without VCI on either the BMET or MoCA, after controlling for age and sex (Table 3). Lacune count was significantly higher in those with VCI, as defined by the BMET and MoCA (Table 3). This remained significant in the sensitivity analysis (BMET: *p* = 0.003, MoCA: *p* = 0.03).

**Table 3:**
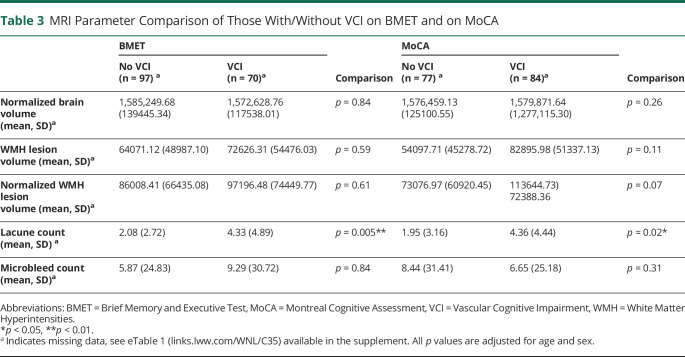
MRI Parameter Comparison of Those With/Without VCI on BMET and on MoCA

On logistic regression, including WMH lesion volume, lacunes, brain volume, age, and sex controlling, only lacune count (OR: 1.63, 95% CI [1.10, 2.41], *p =* 0.014) was a significant predictor of VCI on the BMET (Table 4). This remained significant on the sensitivity analysis: *p* = 0.01. Similarly, on multivariate analysis, only lacune count was a significant predictor of total BMET score (Beta = −0.27, 95% CI [−1.36, −0.19], *p* = 0.01). On multivariate analysis, only lacune count (Beta = −0.28, 95% CI [−0.78, −0.12], *p* = 0.008) predicted performance on the Orientation and Memory subscale of the BMET. These relationships remained significant on the sensitivity analyses: *p* = 0.009 and *p* = 0.008. On multivariate analysis, there were no significant MRI predictors associated with the Executive Functioning and Processing Speed scale, total MoCA score, or increased VCI on the MoCA.

**Table 4:**
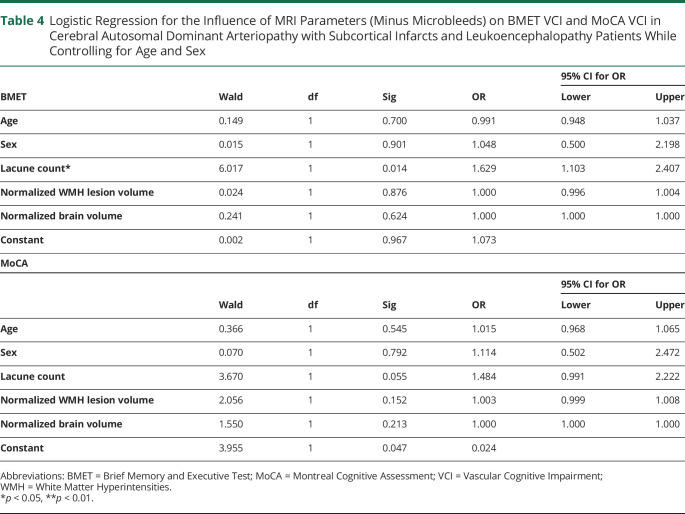
Logistic Regression for the Influence of MRI Parameters (Minus Microbleeds) on BMET VCI and MoCA VCI in Cerebral Autosomal Dominant Arteriopathy with Subcortical Infarcts and Leukoencephalopathy Patients While Controlling for Age and Sex

Only a subgroup of patients with CADASIL had GE/SWI sequences allowing CMB identification (n = 105), and therefore, CMB were not included in the multivariate analysis. However, when controlling for age and sex in a separate analysis, CMB was not associated with VCI on either BMET (*p* = 0.91) or on MoCA (*p* = 0.07). Only age predicted VCI on the MoCA (OR 1.10, 95% CI [1.02, 1.20] *p* = 0.02).

## Discussion

In our group of patients with symptomatic CADASIL, with a mean age of 50 years, we found VCI was present in almost a half of them. Consistent with previous reports in sporadic cerebral small vessel disease and CADASIL, the most prominent deficits were found in executive function and processing speed,^9-14^ although impairment was seen across all aspects of cognition. The most important factor determining cognition was the presence of strokes, and both symptomatic and asymptomatic MRI determined lacunar infarcts.

Most previous studies of cognition and VCI in CADASIL have been in small groups. Consistent with our study, the most common and earliest deficits have been reported in executive function and processing speed.^9-14^ There have been conflicting reports as to whether memory is also affected particularly early in the disease.^5,9,12,13^ Our results demonstrate that it is not spared, with patients with CADASIL performing below controls on memory tasks.^16^ *Z* scores of the 5-item memory repetition task on the BMET were higher than those of the 5-item memory recall task. Because the repetition task requires initial encoding of material in memory, including use of working memory, this is consistent with this ability being spared later into the progression of CADASIL, while long-term memory recall is not.^5,13^

By studying a larger group than those in previous publications, we were able to investigate factors underlying cognitive impairment and VCI. Although vascular risk factors were more common in patients with CADASIL compared with controls, there was no association between them and the risk of VCI. The major clinical determinant of VCI was the presence of a previous stroke, with stroke being associated with an approximately doubling of VCI prevalence, as determined on the BMET, after controlling for age and sex. This finding was consistent with a strong association between the presence, and number, of lacunes with both VCI and the degree of cognitive impairment.^14,22,23^ Such associations with stroke and lacune count remained significant in the sensitivity analyses. This was in contrast to other radiologic features of SVD, NBV, WMH, and CMB, none of which were associated with cognitive impairment. The extent of WMH has been associated with cognitive impairment in some^24^ but not all studies in SVD.^22,23,25^ The lack of association in the CADASIL group may reflect the fact that almost all patients had relatively severe WMH. Nevertheless, the clinical and radiologic results emphasize the key role of stroke and asymptomatic lacunar infarction in precipitating VCI in patients with CADASIL. Therefore, any strategies that could reduce the effect of stroke would have an impact on reducing VCI in such patients.

Recently, *NOTCH3* mutation site has been associated with the severity of CADASIL, particularly age at onset of stroke.^8^ Mutations in the more proximal part of the gene in EGFr 1–6 have been found to be associated with more severe disease.^8^ However, we found no association between EGFr and VCI. It is possible that the lack of a relationship was observed because of most of our sample falling in the EGFr 1–6 grouping (73.3%); future research should investigate the relationship in a more diverse range of mutations.

We assessed cognition with both the BMET and the MoCA, and although the overall prevalence of VCI was broadly similar when defined by the 2 cognitive scales, the associations with MRI parameters varied. When defined by the BMET, the number of lacunes was a strong predictor of VCI, but other MRI parameters were not predictors. When defined by MoCA, lacunes did not predict VCI. This is likely to reflect the differing cognitive domains assessed by each test. The BMET has been designed to be particularly sensitive to impairment in executive function and processing speed seen in SVD,^14,15,19^ whereas MoCA was developed as a global cognitive score primarily to assess cortical dementias.^21^ It has been demonstrated that lacunes impair cognition by disrupting white matter tracts involved in domains such as executive function and processing speed,^24,25^ and therefore, the BMET would be expected to be more sensitive to the detection of this association.^14,15,19^ These differences emphasize the complexity of determining associations between brain structure and cognition and that differing results may be obtained with cognitive batteries tapping into different cognitive domains.

In addition, we saw the prevalence of VCI on the MoCA was higher than that on the BMET. Given the body of research suggesting that the MoCA cutoffs should be lower,^26^ we conducted additional analyses that showed, when using cutoffs of 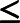
24 and 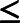
23, VCI on the MoCA to be 37.5%, and 28.24%, respectively. This difference in the prevalence of VCI, on 1 test alone, again emphasizes the importance of using several cognitive tests to sensitively measure cognitive impairment.

Our study has a number of strengths. It was conducted in a relatively large, prospectively recruited, group of patients with CADASIL, all of whom had standardized cognitive testing during recruitment. Original MRI scans were centrally reviewed to determine the presence of WMH, lacunes, CMB, and brain atrophy. Two cognitive assessments, the BMET and MoCA, were performed. However, it also has limitations. Clinical MRI scans were used and performed on different scanners, which may have reduced our sensitivity to detect associations with MRI markers of CADASIL. Education was only partially recorded in the CADASIL population and, therefore, was included as a sensitivity analysis. Education is a protective factor against cognitive decline and VCI in other populations^27,28^; thus, future research should aim to include education as a covariate in the main analyses.

Our study found VCI to be present in 40%–50% of CADASIL patients with a mean age of 50 years. Reductions in cognitive performance were seen across all cognitive domains, including memory. Stroke and lacune count on MRI were both independent predictors of VCI on the BMET. We found no association of VCI with mutation site.

## Glossary

BET: Brief Memory and Executive Test
CADASIL: Cerebral Autosomal Dominant Arteriopathy with Subcortical Infarcts and Leukoencephalopathy
CMB: cerebral microbleed
EGFr: Epidermal Growth Factor–like repeats
GE: Gradient Echo
MoCA: Montreal Cognitive Assessment
NBV: normalised total brain volume
SVD: Small Vessel Disease
SWI: Susceptibility-Weighted Imaging
VCI: vascular cognitive impairment
WMH: White Matter Hyperintensities
